# Reproductive life events and biological aging in women over 50: evidence from DNA methylation clocks

**DOI:** 10.1038/s41514-026-00394-6

**Published:** 2026-05-13

**Authors:** Ya-Qian Xu, Jinming Fu, Chongyu Ding, Hui Zhang, Yulu Gong, Darong Hao, Xiao Sun, Ping Zeng, Xiangwei Li

**Affiliations:** 1 School of Global Health, Chinese Centre for Tropical Diseases Research, Shanghai Jiao Tong University School of Medicine, Shanghai, China; 2 Hainan International Medical Center, Shanghai Jiao Tong University School of Medicine, Qionghai, China; 3 Department of Biostatistics, Jiangsu Engineering Research Center of Biological Data Mining and Healthcare Transformation, Xuzhou Medical University, Xuzhou, China; 4 Jiangsu Key Laboratory of Geriatric Precision Medicine and Aging Intervention, Xuzhou Medical University, Shanghai, China; 5 School of Public Health, Shanghai Jiao Tong University School of Medicine, Shanghai, China; 6 Nanyang Centre for Public Administration, Nanyang Technology University, Singapore, Singapore

**Keywords:** Biomarkers, Diseases, Genetics, Health care, Medical research, Risk factors

## Abstract

As global aging intensifies, elucidating reproductive health’s epigenetic aging associations is crucial. However, the impact of reproductive history on multi-generational DNA methylation (DNAm) clocks remains insufficiently characterized, representing a significant gap in understanding biological vs. chronological aging divergence. This study analyzed 1117 U.S. women aged 50 or older from NHANES 1999–2002 to examine associations between reproductive history and 12 DNA methylation (DNAm) algorithms. Multivariable linear/logistic regression models assessed associations between reproductive indicators and DNAm age acceleration, adjusting for sociodemographic/biological confounders. The *β* coefficients represent the change in DNAm age acceleration in years. Pregnancy frequency (per additional pregnancy) showed positive associations with PhenoAgeacc (*β* = 0.14, 95%CI:0.01–0.27) and GrimAge2Mortacc (*β* = 0.10, 0.03–0.18). Categorical analyses revealed that high pregnancy parity (≥5) significantly increased the odds of GrimAge2Mortacc (OR = 2.34, 95% CI: 1.15–4.71), while high live birth parity (≥5) was associated with increased odds of HannumAgeacc (OR = 2.57, 95% CI: 1.01–6.92). Most primary associations involving second-generation clocks remained robust after false discovery rate correction. While menarche timing showed no significant associations, later menopause and a longer reproductive lifespan were significantly associated with decelerated biological aging after multiple testing correction. These findings suggest that parity and reproductive timing are associated with biological aging trajectories through methylation mechanisms, advancing our understanding of sex-specific aging drivers.

**Figure:**
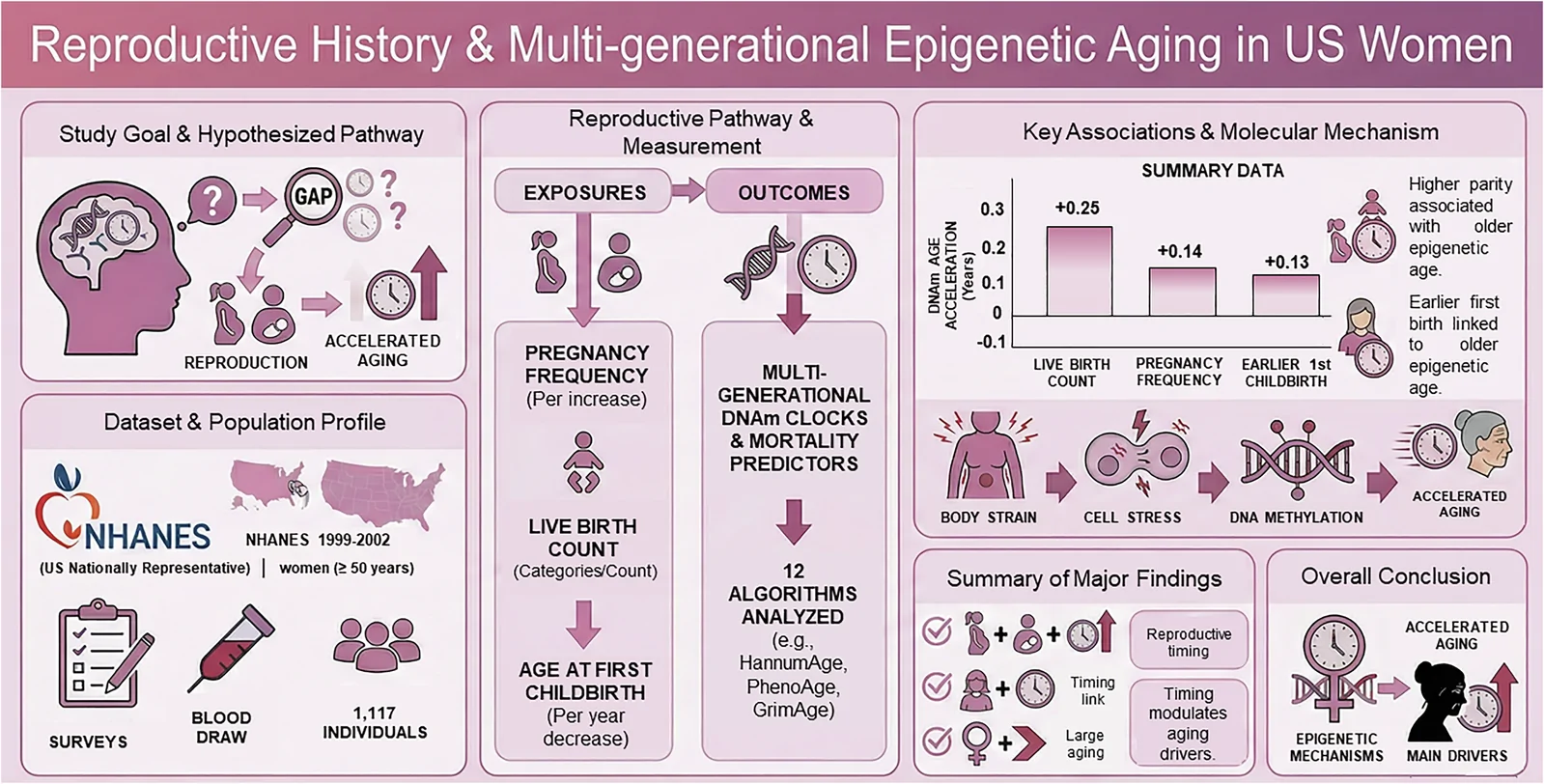

## Introduction

As the global population ages, the effects of aging have emerged as a pressing public health issue, with profound implications for both public health and the socio-economic landscape^1,2^. The relationship between aging and the onset of various diseases has been extensively documented, making the precise identification of biological age a critical focus of contemporary research^3^.

In recent years, a variety of biomarkers for assessing biological aging have been developed, including those based on telomere length and alterations in DNA methylation (DNAm)^4,5^. DNAm, influenced by intrinsic and extrinsic factors, has emerged as a reliable indicator of biological age^6^. Prominent biomarkers, including HorvathAge^7^, HannumAge^8^, SkinBloodAge^9^, PhenoAge^10^, and GrimAge^11^, have shown strong correlations with biological age^12,13^. The acceleration of these biomarkers, as reflected by the discrepancy between biological and chronological age, serves as a potent predictor of mortality and age-related diseases^14,15^.

Instead, evidence suggests that reproductive life events can leave lasting epigenetic marks. For instance, research in younger cohorts has shown that higher parity is associated with approximately 0.5 years of DNAm age acceleration per pregnancy^16^. However, findings regarding early-life milestones like menarche or lifetime menstrual duration remain inconsistent and have been insufficiently explored in older, post-menopausal cohorts where the cumulative impact of reproductive load is fully realized^17,18^. Women’s unique biological characteristics, particularly reproductive events such as the age of menarche, menopause, lifetime menstrual duration, and number of pregnancies, have been shown to be associated with a range of diseases, including atrial fibrillation^19^, metabolic syndrome^20^, and depression^21^. Given that reproductive events are closely associated with hormonal fluctuations, immune function, and metabolic pathways, they may modulate DNAm patterns through epigenetic mechanisms^22,23^. Consequently, reproductive history may play a critical role in the aging process through the “weathering” of biological systems^24–28^.

This study seeks to bridge the existing research gap by examining the associations between primary reproductive exposures (specifically parity, age at first birth, age at menarche, and total menstrual years) and 12 commonly used DNAm algorithms. Using data from the US National Health and Nutrition Examination Survey (NHANES) for women over the age of 50, we hypothesized that higher parity and earlier reproductive timing would be associated with greater DNAm age acceleration, particularly in second-generation clocks (e.g., PhenoAge and GrimAge) that are sensitive to physiological dysregulation^10,11^.

## Results

### Study participants

Table 1 presents the characteristics of the study population, consisting of 1117 female participants. The average age (SD) of the participants was 65.37 (9.82) years and 65.92 (10.22) years in the 1999–2000 cycle and 2001–2002 cycle, respectively. The distributions of race/ethnicity, educational levels, BMI, smoking status, and alcohol consumption were comparable between the two subsets. These variables were included as covariates in multivariable models to mitigate potential confounding, including confounding by indication for hormone therapy.

**Table 1:** Characteristics of the study female population

Characteristics	NHANES 1999–2000 *N* = 526	NHANES 2001–2002 *N* = 591	Total *N* = 1117
Age (years; mean ± SD)	65.37 ± 9.82	65.92 ± 10.22	65.66 ± 10.03
Educational levels (*N*/%) ^a,b,c^			
Low (≤9 years)	170 (32.38)	121 (20.54)	291 (26.12)
Intermediate (10–11 years)	117 (22.29)	106 (18.00)	223 (20.02)
High (≥12 years)	238 (45.33)	362 (61.46)	600 (53.86)
Body mass index (*N*/%)			
Underweight (<18·5 kg/m^2^)	4 (0.76)	5 (0.85)	9 (0.81)
Normal weight (18·5- < 25·0 kg/m^2^)	137 (26.05)	158 (26.73)	295 (26.41)
Overweight (25·0- < 30·0 kg/m^2^)	157 (29.85)	190 (32.15)	347 (31.07)
Obesity (≥30·0 kg/m^2^)	228 (43.35)	238 (40.27)	466 (41.72)
Smoking status (*N*/%)^d,e,f^			
Never smoker	323 (61.64)	348 (59.08)	671 (60.29)
Former smoker	133 (25.38)	166 (28.18)	299 (26.86)
Current smoker	68 (12.98)	75 (12.73)	143 (12.85)
Alcohol consumption (*N*/%)			
Never	146 (29.55)	150 (27.12)	296 (28.27)
Ever	134 (27.13)	145 (26.22)	279 (26.65)
Current	214 (43.32)	258 (46.65)	472 (45.08)
Race/ethnicity, *N* (%)			
Mexican American	177 (33.65)	134 (22.67)	311 (27.84)
Other Hispanic	49 (9.32)	27 (4.57)	76 (6.80)
Non-Hispanic White	181 (34.41)	274 (46.36)	455 (40.73)
Non-Hispanic Black	100 (19.01)	132 (22.34)	232 (20.77)
Other races-including multi-racial	19 (3.61)	24 (4.06)	43 (3.85)

*SD* standard deviation.

^a^1999–2000 Data missing for 1 participant.

^b^2001–2002 Data missing for 2 participants.

^c^1999–2002 Data missing for 3 participants.

^d^1999–2000 Data missing for 2 participants.

^e^1999–2000 Data missing for 2 participants.

^f^1999–2002 Data missing for 4 participants.

As shown in Table S1, participants aged 50–65 years exhibited significantly lower mean values for most original DNAm algorithms compared to those aged ≥65 years, with the exception of DunedinPoAm and HorvathTelo. In contrast, the levels of accelerated versions, HorvathAgeacc, HannumAgeacc, SkinBloodAgeacc, PhenoAgeacc, LinAgeacc, WeidnerAgeacc, VidalBraloAgeacc, ZhangAgeacc, GrimAgeMortacc, and GrimAge2Mortacc were higher among participants aged 50–65 years.

The reproductive history revealed that 93% of participants had been pregnant at least once, with an average of 4.0 (SD 2.5) pregnancies and 3.0 (SD 1.8) live births per woman. The mean age at first live birth was 21.0 (SD 4.2) years, while the average age at last live birth occurred at 31.0 (SD 6.5) years, and 55.8% of women reported breastfeeding their infants. Regarding menstrual and hormonal history, 43.7% of participants reported exogenous female hormone use, and 44.8% reported oral contraceptive use. Women experienced menarche at a mean age of 12.9 (SD 1.7) years and reached menopause at 45.3 (SD 8.2) years, resulting in an average reproductive lifespan of 32.4 (SD 8.2) years among those aged 50 years or older.

### Reproductive history and DNAm algorithms

Table 2 summarizes the associations between reproductive history variables with DNAm algorithms as continuous variables. The number of pregnancies was positively associated with PhenoAgeacc (*β* = 0.14, 95% CI: 0.01–0.27; *P* = 0.040, *P*_FDR_ > 0.05) and GrimAge2Mortacc (*β* = 0.10, 95% CI: 0.03–0.18; *P* = 0.007, *P*_FDR_ < 0.05), while demonstrating an inverse relationship with VidalBraloAgeacc (*β* = −0.15, 95% CI: −0.24 to −0.05; *P* = 0.003, *P*_FDR_ < 0.05). These results suggest that different algorithms may capture distinct biological responses to reproductive load. The number of live births was significantly associated with five DNAm algorithms, showing particularly strong positive associations with PhenoAgeacc (*β* = 0.25, 95% CI: 0.08–0.41; *P* = 0.004, *P*_FDR_ < 0.05), HannumAgeacc (*β* = 0.20, 95% CI: 0.06–0.33; *P* = 0.005, *P*_FDR_ < 0.05), GrimAge2Mortacc (*β* = 0.17, 95% CI: 0.08–0.27; *P* < 0.001, *P*_FDR_ < 0.05), and DunedinPoAm (*β* = 0.01, 95% CI: 0.00–0.02; *P* = 0.015, *P*_FDR_ < 0.05), and a modest negative association with HorvathTelo (*β* = −0.01, 95% CI: −0.02–0.00; *P* = 0.016, *P*_FDR_ < 0.05). Earlier age at first live birth predicted greater epigenetic aging, with the strongest effects observed for PhenoAgeacc (*β* = −0.13, 95% CI: −0.23 to −0.03; *P* = 0.014, *P*_FDR_ < 0.05), GrimAge2Mortacc (*β* = −0.12, 95% CI: −0.18 to −0.07; *P* < 0.001, *P*_FDR_ < 0.05), HannumAgeacc (*β* = −0.09, 95% CI: −0.17 to −0.01; *P* = 0.039, *P*_FDR_ > 0.05), SkinBloodAgeacc (*β* = −0.09, 95% CI: −0.16–0.01; *P* = 0.031, *P*_FDR_ > 0.05), and GrimAgeMortacc (*β* = −0.08, 95% CI: −0.13 to −0.03; *P* = 0.002, *P*_FDR_ < 0.05), and a modest association with DunedinPoAm (*β* = −0.01, 95% CI: −0.02–0.00; *P* < 0.001, *P*_FDR_ < 0.05). No significant associations were found for ever having been pregnant, age at last live birth, or breastfeeding history.

**Table 2:** Associations of DNAm algorithms with reproductive history

Women health	Model 1		Model 2	
	Coefficient (95% CI)^a^	*P*-value	Coefficient (95% CI)^a^	*P*-value
Ever been pregnant				
HorvathAgeacc	0.66 (−0.64, 1.96)	0.317	0.82 (−0.49, 2.13)	0.218
HannumAgeacc	−0.23 (−1.50, 1.03)	0.717	−0.07 (−1.34, 1.21)	0.920
SkinBloodAgeacc	0.05 (−1.11, 1.22)	0.927	0.19 (−1.10, 1.38)	0.761
PhenoAgeacc	−0.22 (−1.77, 1.34)	0.783	−0.14 (−1.72, 1.38)	0.867
LinAgeacc	0.66 (−1.08, 2.40)	0.455	0.49 (−1.30, 2.27)	0.592
WeidnerAgeacc	1.20 (−0.79, 3.19)	0.237	1.11 (−0.91, 3.12)	0.281
VidalBraloAgeacc	1.03 (−0.09, 2.14)	0.070	1.02 (−0.11, 2.15)	0.076
ZhangAgeacc	0.00 (−0.42, 0.43)	0.987	0.03 (−0.41, 0.47)	0.887
GrimAgeMortacc	−0.89 (−1.83, 0.05)	0.064	−0.60 (−1.38, 0.18)	0.131
GrimAge2Mortacc	−0.95 (−1.98, 0.09)	0.072	−0.64 (−1.53, 0.24)	0.154
DunedinPoAm	−0.01 (−0.03, 0.01)	0.315	−0.01 (−0.02, 0.01)	0.523
HorvathTelo	0.04 (−0.01, 0.10)	0.111	0.04 (−0.02, 0.09)	0.197
Number of pregnant times				
HorvathAgeacc	−0.01 (−0.12, 0.10)	0.857	−0.02 (−0.13, 0.10)	0.793
HannumAgeacc	0.10 (−0.01, 0.21)	0.065	0.10 (−0.01, 0.20)	0.087
SkinBloodAgeacc	0.03 (−0.07, 0.13)	0.508	0.03 (−0.07, 0.13)	0.590
PhenoAgeacc	0.16 (0.02, 0.29)	0.022	0.14 (0.01, 0.27)	0.040
LinAgeacc	−0.02 (−0.17, 0.13)	0.841	−0.03 (−0.18, 0.12)	0.723
WeidnerAgeacc	−0.09 (−0.26, 0.08)	0.318	−0.08 (−0.26, 0.09)	0.336
VidalBraloAgeacc	−0.15 (−0.25, −0.06)	0.001	−0.15 (−0.24, −0.05)	0.003
ZhangAgeacc	0.01 (−0.02, 0.05)	0.478	0.01 (−0.03, 0.05)	0.516
GrimAgeMortacc	0.02 (−0.06, 0.11)	0.555	0.03 (−0.04, 0.09)	0.462
GrimAge2Mortacc	0.11 (0.02, 0.20)	0.0131	0.10 (0.03, 0.18)	0.007
DunedinPoAm	0.00 (0.00, 0.00)	0.120	0.01 (−0.01, 0.02)	0.120
HorvathTelo	0.00 (0.00, 0.00)	0.070	0.01 (−0.01, 0.02)	0.065
Number of live births				
HorvathAgeacc	0.07 (−0.07, 0.21)	0.303	0.07 (−0.07, 0.21)	0.299
HannumAgeacc	0.20 (0.06, 0.34)	0.003	0.20 (0.06, 0.33)	0.005
SkinBloodAgeacc	0.13 (0.00, 0.26)	0.043	0.13 (0.00, 0.26)	0.053
PhenoAgeacc	0.26 (0.09, 0.43)	0.002	0.25 (0.08, 0.41)	0.004
LinAgeacc	0.05 (−0.14, 0.23)	0.611	0.03 (−0.16, 0.22)	0.744
WeidnerAgeacc	0.04 (−0.18, 0.25)	0.742	0.03 (−0.18, 0.24)	0.784
VidalBraloAgeacc	−0.08 (−0.20, 0.03)	0.164	−0.08 (−0.20, 0.05)	0.222
ZhangAgeacc	0.04 (−0.01, 0.09)	0.084	0.04 (−0.01, 0.09)	0.092
GrimAgeMortacc	0.03 (−0.07, 0.13)	0.577	0.07 (−0.02, 0.15)	0.130
GrimAge2Mortacc	0.15 (0.04, 0.26)	0.008	0.17 (0.08, 0.27)	<0.001
DunedinPoAm	0.01 (−0.01,0.03)	0.075	0.01 (0.00,0.02)	0.015
HorvathTelo	−0.01 (−0.02, 0.00)	0.031	−0.01 (−0.02, 0.00)	0.016
Age at first live birth				
HorvathAgeacc	−0.01 (−0.09, 0.08)	0.988	0.01 (−0.08, 0.09)	0.886
HannumAgeacc	−0.09 (−0.18, −0.01)	0.028	−0.09 (−0.17, −0.01)	0.039
SkinBloodAgeacc	−0.09 (−0.16, −0.01)	0.027	−0.09 (−0.16, 0.01)	0.031
PhenoAgeacc	−0.15 (−0.25, −0.05)	0.004	−0.13 (−0.23, −0.03)	0.014
LinAgeacc	−0.03 (−0.14, 0.09)	0.667	−0.02 (−0.13, 0.10)	0.792
WeidnerAgeacc	0.09 (−0.04, 0.22)	0.177	0.08 (−0.05, 0.21)	0.242
VidalBraloAgeacc	0.05 (−0.02, 0.13)	0.154	0.05 (−0.02, 0.13)	0.164
ZhangAgeacc	−0.02 (−0.04, 0.01)	0.233	−0.02 (−0.05, 0.01)	0.244
GrimAgeMortacc	−0.12 (−0.19, −0.06)	<0.001	−0.08 (−0.13, −0.03)	0.002
GrimAge2Mortacc	−0.17 (−0.24, −0.11)	<0.001	−0.12 (−0.18, −0.07)	<0.001
DunedinPoAm	−0.01 (−0.02, 0.00)	<0.001	−0.01 (−0.02, 0.00)	<0.001
HorvathTelo	0.01 (−0.01, 0.03)	0.156	0.01 (−0.01, 0.03)	0.266
Age at last live birth				
HorvathAgeacc	−0.01 (−0.06, 0.05)	0.928	0.01 (−0.01, 0.03)	0.898
HannumAgeacc	−0.01 (−0.06, 0.05)	0.929	0.01 (−0.05, 0.06)	0.935
SkinBloodAgeacc	−0.01 (−0.06, 0.04)	0.733	−0.01 (−0.06, 0.05)	0.834
PhenoAgeacc	0.01 (−0.06, 0.07)	0.813	0.02 (−0.05, 0.08)	0.618
LinAgeacc	−0.06 (−0.13, 0.01)	0.104	−0.06 (−0.14, 0.01)	0.094
WeidnerAgeacc	0.01 (−0.08, 0.09)	0.942	−0.01 (−0.09, 0.08)	0.902
VidalBraloAgeacc	−0.03 (−0.08, 0.02)	0.244	−0.02 (−0.07, 0.02)	0.334
ZhangAgeacc	0.01 (−0.02, 0.02)	0.823	0.01 (−0.02, 0.02)	0.733
GrimAgeMortacc	−0.05 (−0.09, 0.01)	0.008	−0.02 (−0.05, 0.02)	0.336
GrimAge2Mortacc	−0.40 (−0.08, 0.01)	0.111	0.00 (−0.04, 0.04)	0.970
DunedinPoAm	−0.01 (−0.02, 0.00)	0.129	0.00 (−0.01, 0.01)	0.967
HorvathTelo	0.01 (−0.01, 0.03)	0.697	0.00 (−0.01, 0.01)	0.877
Breastfed				
HorvathAgeacc	0.53 (−0.17, 1.22)	0.136	0.46 (−0.24, 1.16)	0.199
HannumAgeacc	−0.09 (−0.77, 0.60)	0.805	−0.15 (−0.84, 0.54)	0.666
SkinBloodAgeacc	0.28 (−0.35, 0.91)	0.387	0.24 (−0.40, 0.87)	0.467
PhenoAgeacc	−0.30 (−1.14, 0.54)	0.479	−0.39 (−1.24, 0.45)	0.358
LinAgeacc	0.62 (−0.31, 1.55)	0.192	0.67 (−0.27, 1.62)	0.160
WeidnerAgeacc	−0.17 (−1.23, 0.89)	0.749	−0.08 (−1.15, 0.99)	0.883
VidalBraloAgeacc	0.24 (−0.35, 0.84)	0.423	0.17 (−0.43, 0.77)	0.584
ZhangAgeacc	0.14 (−0.10, 0.37)	0.250	0.12 (−0.11, 0.35)	0.308
GrimAgeMortacc	0.72 (0.22, 1.22)	0.005	0.23 (−0.19, 0.64)	0.286
GrimAge2Mortacc	0.49 (−0.06, 1.05)	0.080	0.04 (−0.43, 0.52)	0.864
DunedinPoAm	0.01 (−0.01, 0.02)	0.162	0.00 (−0.01, 0.01)	0.930
HorvathTelo	0.01 (−0.03, 0.03)	0.908	0.01 (−0.02, 0.04)	0.444

Model 1, adjusted for age, leukocyte composition (CD8TPP, CD4TPP, Nkcell, Bcell, MonoPP, NeuPP).

Model 2, similar as model 1, additionally adjusted for BMI, education, annual household income, smoking status (never smoker, former smoker, current smoker), and drinking status (never, ever, current).

*CI* confidence interval.

**Fig. 1:**
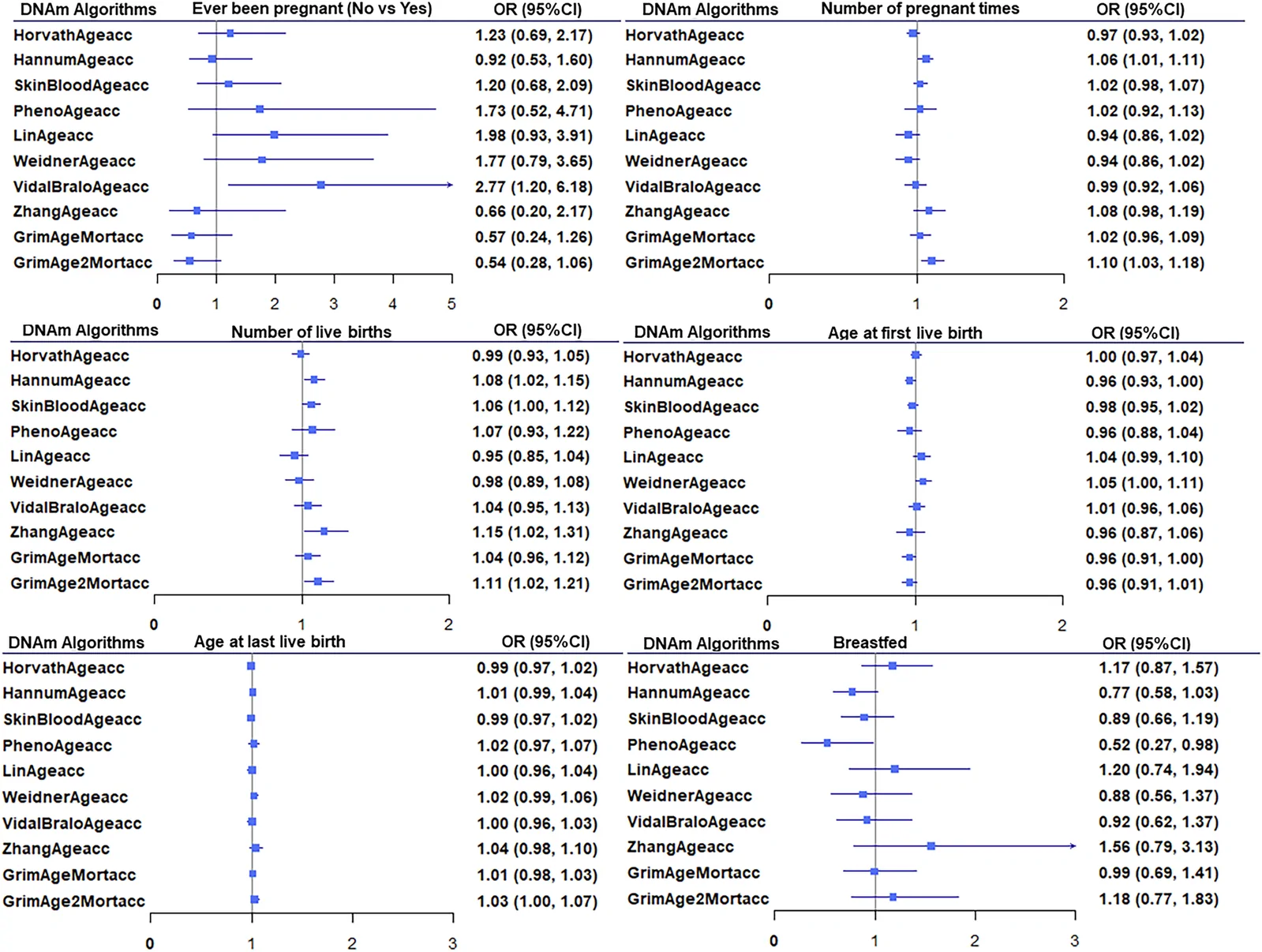
Associations of DNAm age acceleration with reproductive history. The forest plots display odds ratios (ORs) and 95% confi dence intervals (CIs) for the association of various reproductive history factors with ten epigenetic age acceleration algorithms.

When examining categorical associations (Fig. 1), women with a history of pregnancy had 2.77-fold increased odds (95% CI: 1.20–6.18, *P*_FDR_ > 0.05) of advanced biological aging by VidalBraloAgeacc. Each additional pregnancy conferred 6% greater odds of HannumAge acceleration (OR = 1.06, 95% CI: 1.01–1.11, *P*_FDR_ < 0.05) and 10% greater odds of GrimAge2Mortacc (OR = 1.10, 95% CI: 1.03–1.18, *P*_FDR_ < 0.05). Live birth parity showed dose-dependent relationships, with each birth associated with 8−15% increased odds of accelerated aging across multiple algorithms (HannumAgeacc OR = 1.08, 95% CI: 1.02−1.15, *P*_FDR_ < 0.05; ZhangAgeacc OR = 1.15, 95% CI: 1.02–1.31, *P*_FDR_ > 0.05; GrimAge2Mortacc OR = 1.11, 95% CI: 1.02−1.21, *P*_FDR_ < 0.05). Furthermore, each additional year of age at last live birth was associated with 3% higher odds of GrimAge2 acceleration (OR = 1.03; 95% CI: 1.00−1.07, *P*_FDR_ > 0.05). Although the individual linear coefficients were modest, the associations, particularly those for mortality-related algorithms, remained robust after FDR correction. This pattern is consistent with a cumulative impact of reproductive history on biological aging, although the magnitude of the effect should be interpreted cautiously.

Threshold analysis (Table 3) showed women with ≥5 live births had 2.57-fold increased odds (95% CI: 1.01–6.92; *P*-trend = 0.002) of HannumAge acceleration, while those with ≥5 pregnancies had 2.34-fold increased odds (1.15–4.71; *P*-trend = 0.010) of GrimAge2Mortacc acceleration. High parity (≥5 pregnancies) was associated with reduced odds of LinAge acceleration (OR = 0.43, 0.20−0.93). VidalBraloAgeacc showed progressively decreasing odds with increasing pregnancy number. Eight other DNA methylation aging measures showed no significant associations with parity measures (Supplementary Tables S2–S6).

**Table 3:** Odds ratio of five DNAm algorithms with the number of pregnant times and the number of live births

Women health		HannumAgeacc		GrimAgeMortacc		GrimAge2Mortacc		LinAgeacc		VidalBraloAgeacc	
		Model 1	Model 2	Model 1	Model 2	Model 1	Model 2	Model 1	Model 2	Model 1	Model 2
	Cases (*n*/*N*)	OR (95% CI)	OR (95% CI)	OR (95% CI)	OR (95% CI)	OR (95% CI)	OR (95% CI)	OR (95% CI)	OR (95% CI)	OR (95% CI)	OR (95% CI)
Number of pregnant times	1043										
Zero	73	Ref	Ref	Ref	Ref	Ref	Ref	Ref	Ref	Ref	Ref
One	79	1.21 (0.58, 2.49)	1.17 (0.56, 2.43)	1.37 (0.56, 3.34)	1.15 (0.41, 3.26)	1.59 (0.66, 3.87)	1.35 (0.54, 3.40)	0.52 (0.19, 1.45)	0.50 (0.17, 1.40)	0.41 (0.14, 1.16)	0.39 (0.13, 1.12)
Two	142	0.71 (0.37, 1.34)	0.70 (0.37, 1.33)	2.00 (0.91, 4.43)	1.99 (0.81, 5.19)	2.06 (0.92, 4.64)	1.73 (0.75, 3.99)	0.57 (0.24, 1.36)	0.55 (0.22, 1.34)	0.36 (0.14, 0.90)	0.34 (0.13, 0.87)
Three	190	0.90 (0.49, 1.66)	0.90 (0.49, 1.66)	1.62 (0.75, 3.50)	1.74 (0.73, 4.44)	1.36 (0.65, 2.83)	1.29 (0.61, 2.73)	0.50 (0.22, 1.15)	0.50 (0.21, 1.18)	0.26 (0.11, 0.66)	0.25 (0.10, 0.64)
Four	176	1.36 (0.73, 2.52)	1.33 (0.72, 2.47)	1.78 (0.83, 3.85)	1.85 (0.77, 4.71)	1.30 (0.61, 2.75)	1.25 (0.57, 2.72)	0.52 (0.23, 1.19)	0.53 (0.23, 1.23)	0.57 (0.24, 1.37)	0.55 (0.23, 1.33)
≥Five	383	1.37 (0.78, 2.41)	1.32 (0.75, 2.33)	2.13 (1.04, 4.39)	2.02 (0.89, 4.93)	2.35 (1.18, 4.67)	2.34 (1.15, 4.71)	0.44 (0.21, 0.93)	0.43 (0.20, 0.93)	0.37 (0.16, 0.84)	0.35 (0.15, 0.82)
*P*-trend		0.016	0.026	0.045	0.114	0.020	0.010	0.100	0.095	0.349	0.306
Number of live births	970										
Zero	26	Ref	Ref	Ref	Ref	Ref	Ref	Ref	Ref	Ref	Ref
One	106	1.90 (0.69, 5.24)	1.76 (0.65, 5.02)	0.37 (0.13, 1.05)	0.33 (0.10, 1.05)	1.18 (0.35, 3.93)	1.00 (0.26, 3.50)	0.53 (0.12, 2.30)	0.50 (0.12, 2.54)	2.75 (0.46, 16.40)	2.68 (0.49, 18.26)
Two	210	1.41 (0.54, 3.70)	1.36 (0.53, 3.70)	0.51 (0.19, 1.36)	0.58 (0.20, 1.74)	0.96 (0.31, 2.96)	0.96 (0.26, 3.06)	0.89 (0.24, 3.30)	0.87 (0.26, 3.98)	2.43 (0.43, 13.85)	2.37 (0.46, 15.48)
Three	198	2.03 (0.77, 5.34)	1.93 (0.75, 5.27)	0.59 (0.22, 1.56)	0.63 (0.21, 1.90)	0.95 (0.31, 2.91)	1.11(0.30, 3.56)	0.76 (0.20, 2.88)	0.74 (0.21, 3.45)	2.72 (0.47, 15.66)	2.65 (0.51, 17.49)
Four	140	2.64 (0.98, 7.11)	2.49 (0.94, 6.94)	0.59 (0.22, 1.61)	0.67 (0.22, 2.08)	0.74 (0.23, 2.34)	0.78 (0.21, 2.60)	0.53 (0.13, 2.13)	0.52 (0.13, 2.53)	2.07 (0.35, 12.18)	2.01 (0.37, 13.55)
≥Five	290	2.76 (1.07, 7.14)	2.57 (1.01, 6.92)	0.65 (0.25, 1.68)	0.71 (0.25, 2.08)	1.53 (0.51, 4.63)	1.79 (0.50, 5.67)	0.64 (0.17, 2.38)	0.61 (0.18, 2.82)	2.62 (0.46, 15.01)	2.53 (0.49, 16.77)
*P*-trend		0.001	0.002	0.219	0.162	0.166	0.042	0.511	0.512	0.820	0.871

Model 1, adjusted for age, leukocyte composition (CD8TPP, CD4TPP, Nkcell, Bcell, MonoPP, NeuPP).

Model 2, similar as model 1, additionally adjusted for BMI, education, annual household income, smoking status (never smoker, former smoker, current smoker), and drinking status (never, ever, current).

*OR* odds ratio, *CI* confidence interval.

### Menstrual/hormonal history and DNAm algorithms

Table 4 exhibits the associations of menstrual/hormonal history with DNAm algorithms. Exogenous female hormone use showed divergent associations across epigenetic clocks, with protective effects observed for VidalBraloAgeacc (*β* = −0.64, 95% CI: −1.22 to −0.05; *P* = 0.032, *P*_FDR_ > 0.05), but accelerated aging patterns for both GrimAgeMortacc (*β* = 0.43, 95% CI: 0.03−0.83; *P* = 0.037, *P*_FDR_ < 0.05) and GrimAge2Mortacc (*β* = 0.73, 95% CI: 0.27−1.18; *P* = 0.002, *P*_FDR_ < 0.05). These bidirectional findings suggest that while hormone therapy may associate with certain cellular age markers, it does not uniformly delay physiological aging as measured by mortality-predictive clocks. Oral contraceptive use was associated only with DunedinPoAm (*β* = 0.01, 95% CI: 0.00−0.02; *P* = 0.034, *P*_FDR_ > 0.05). Menopausal timing was linked to slower aging by VidalBraloAgeacc (*β* = −0.05, 95% CI: −0.09 to −0.01; *P* = 0.010, *P*_FDR_ < 0.05). Similarly, longer reproductive lifespan correlated with decelerated VidalBraloAgeacc (*β* = −0.04, 95% CI: −0.08 to −0.01; *P* = 0.017, *P*_FDR_ < 0.05). No significant associations were found between any DNAm algorithms and age at first menstrual period.

**Table 4:** Associations of DNAm algorithms with menstrual/hormonal history

Women health	Model 1		Model 2	
	Coefficient (95% CI)^a^	*P*-value	Coefficient (95% CI)^a^	*P*-value
Usage of female hormones				
HorvathAgeacc	−0.11 (−0.79, 0.56)	0.743	−0.14 (−0.82, 0.54)	0.682
HannumAgeacc	0.05 (−0.61, 0.71)	0.878	0.01 (−0.66, 0.67)	0.985
SkinBloodAgeacc	−0.01 (−0.61, 0.61)	0.996	−0.04 (−0.65, 0.58)	0.911
PhenoAgeacc	0.82 (0.01, 1.62)	0.047	0.72 (−0.09, 1.53)	0.083
LinAgeacc	0.32 (−0.59, 1.22)	0.489	0.24 (−0.67, 1.16)	0.601
WeidnerAgeacc	−0.77 (−1.78, 0.27)	0.147	−0.76 (−1.80, 0.29)	0.155
VidalBraloAgeacc	−0.67 (−1.25, −0.09)	0.022	−0.64 (−1.22, −0.05)	0.032
ZhangAgeacc	−0.02 (−0.24, 0.21)	0.887	−0.02 (−0.24, 0.20)	0.862
GrimAgeMortacc	0.38 (−0.11, 0.87)	0.123	0.43 (0.03, 0.83)	0.037
GrimAge2Mortacc	0.76 (0.22, 1.30)	0.005	0.73 (0.27, 1.18)	0.002
DunedinPoAm	0.01 (−0.01, 0.02)	0.073	0.01 (0.00, 0.02)	0.062
HorvathTelo	0.01 (−0.02, 0.04)	0.521	0.01 (−0.02, 0.04)	0.570
Usage of birth control pills				
HorvathAgeacc	0.10 (−0.66, 0.87)	0.792	0.07 (−0.70, 0.85)	0.857
HannumAgeacc	0.74 (−0.01, 1.49)	0.051	0.64 (−0.12, 1.39)	0.097
SkinBloodAgeacc	0.34 (−0.35, 1.03)	0.337	0.25 (−0.45, 0.95)	0.482
PhenoAgeacc	0.98 (0.07, 1.90)	0.035	0.87 (−0.05, 1.79)	0.064
LinAgeacc	0.78 (−0.24, 1.80)	0.134	0.67 (−0.37, 1.71)	0.207
WeidnerAgeacc	0.73 (−0.44, 1.90)	0.222	0.73 (−0.45, 1.92)	0.225
VidalBraloAgeacc	0.15 (−0.50, 0.81)	0.648	0.22 (−0.45, 0.88)	0.524
ZhangAgeacc	0.11 (−0.14, 0.36)	0.389	0.09 (−0.17, 0.34)	0.491
GrimAgeMortacc	0.18 (−0.37, 0.74)	0.521	0.32 (−0.13, 0.78)	0.165
GrimAge2Mortacc	0.34 (−0.27, 0.95)	0.276	0.42 (−0.10, 0.94)	0.113
DunedinPoAm	0.01 (−0.01, 0.02)	0.104	0.01 (0.00, 0.02)	0.034
HorvathTelo	−0.01 (−0.04, 0.02)	0.455	−0.01 (−0.04, 0.02)	0.473
Age when first menstrual period				
HorvathAgeacc	0.01 (−0.18,0.21),	0.885	0.03 (−0.16,0.23)	0.751
HannumAgeacc	−0.04 (−0.23,0.15),	0.650	−0.04 (−0.24,0.15)	0.657
SkinBloodAgeacc	−0.03 (−0.21,0.14),	0.699	−0.02 (−0.20,0.16)	0.822
PhenoAgeacc	−0.09 (−0.32,0.15),	0.471	−0.04 (−0.28,0.20)	0.745
LinAgeacc	−0.08 (−0.34,0.18),	0.555	−0.09 (−0.36,0.18)	0.515
WeidnerAgeacc	−0.09 (−0.39,0.21)	0.563	−0.13 (−0.44,0.17)	0.388
VidalBraloAgeacc	−0.11 (−0.28,0.06)	0.198	−0.11 (−0.28,0.06)	0.207
ZhangAgeacc	−0.00 (−0.07,0.06)	0.960	−0.01 (−0.07,0.06)	0.869
GrimAgeMortacc	−0.11 (−0.25,0.03)	0.126	−0.00 (−0.12,0.12)	0.991
GrimAge2Mortacc	−0.12 (−0.28,0.03)	0.128	0.01 (−0.12,0.14)	0.913
DunedinPoAm	−0.00 (−0.00,0.00)	0.551	0.00 (−0.00,0.00)	0.549
HorvathTelo	0.00 (−0.00,0.01)	0.299	0.00 (−0.00,0.01)	0.357
Age at last menstrual period				
HorvathAgeacc	−0.02 (−0.06,0.03)	0.452	−0.01 (−0.06, 0.03)	0.548
HannumAgeacc	−0.01 (−0.05,0.03)	0.559	−0.00 (−0.05, 0.04)	0.883
SkinBloodAgeacc	−0.02 (−0.06,0.01)	0.212	−0.02 (−0.06, 0.02)	0.370
PhenoAgeacc	−0.02 (−0.07,0.03)	0.453	−0.00 (−0.05, 0.05)	0.969
LinAgeacc	−0.02 (−0.07,0.04)	0.554	−0.01 (−0.07, 0.05)	0.784
WeidnerAgeacc	−0.02 (−0.08,0.05)	0.578	−0.02 (−0.09, 0.05)	0.531
VidalBraloAgeacc	−0.05 (−0.08, −0.01)	0.010	−0.05 (−0.09, −0.01)	0.010
ZhangAgeacc	−0.00 (−0.02,0.01)	0.779	0.00 (−0.01, 0.01)	0.987
GrimAgeMortacc	−0.04 (−0.07, −0.01)	0.021	−0.02 (−0.05, 0.00)	0.057
GrimAge2Mortacc	−0.04 (−0.07, −0.01)	0.023	−0.02 (−0.05, 0.00)	0.100
DunedinPoAm	−0.00 (−0.00,0.00)	0.096	−0.00 (−0.00, 0.00)	0.458
HorvathTelo	0.00 (−0.00,0.00)	0.525	0.00 (−0.00, 0.00)	0.655
Menstrual years				
HorvathAgeacc	−0.02 (−0.06, 0.03)	0.468	−0.01 (−0.06, 0.03)	0.535
HannumAgeacc	−0.01 (−0.05, 0.03)	0.646	−0.00 (−0.04, 0.04)	0.898
SkinBloodAgeacc	−0.02 (−0.06, 0.02)	0.261	−0.02 (−0.06, 0.02)	0.424
PhenoAgeacc	−0.01 (−0.07, 0.00)	0.571	0.00 (−0.05, 0.00)	0.949
LinAgeacc	−0.01 (−0.07, 0.00)	0.646	−0.00 (−0.06, 0.00)	0.886
WeidnerAgeacc	−0.01 (−0.08, 0.05)	0.655	−0.02 (−0.08, 0.05)	0.653
VidalBraloAgeacc	−0.04 (−0.08, −0.01)	0.018	−0.04 (−0.08, −0.01)	0.017
ZhangAgeacc	−0.00 (−0.02, 0.01)	0.827	0.00 (−0.01, 0.02)	0.921
GrimAgeMortacc	−0.03 (−0.06, 0.00)	0.051	−0.02 (−0.05, 0.00)	0.057
GrimAge2Mortacc	−0.03 (−0.07, 0.00)	0.050	−0.02 (−0.05, 0.00)	0.089
DunedinPoAm	−0.00 (−0.00, 0.00)	0.126	−0.00 (−0.00, 0.00)	0.380
HorvathTelo	0.00 (−0.00, 0.00)	0.712	0.00 (−0.00, 0.00)	0.823

Model 1, adjusted for age, leukocyte composition (CD8TPP, CD4TPP, Nkcell, Bcell, MonoPP, NeuPP).

Model 2, similar as model 1, additionally adjusted for BMI, education, annual household income, smoking status (never smoker, former smoker, current smoker), and drinking status (never, ever, current).

*CI* confidence interval.

**Fig. 2:**
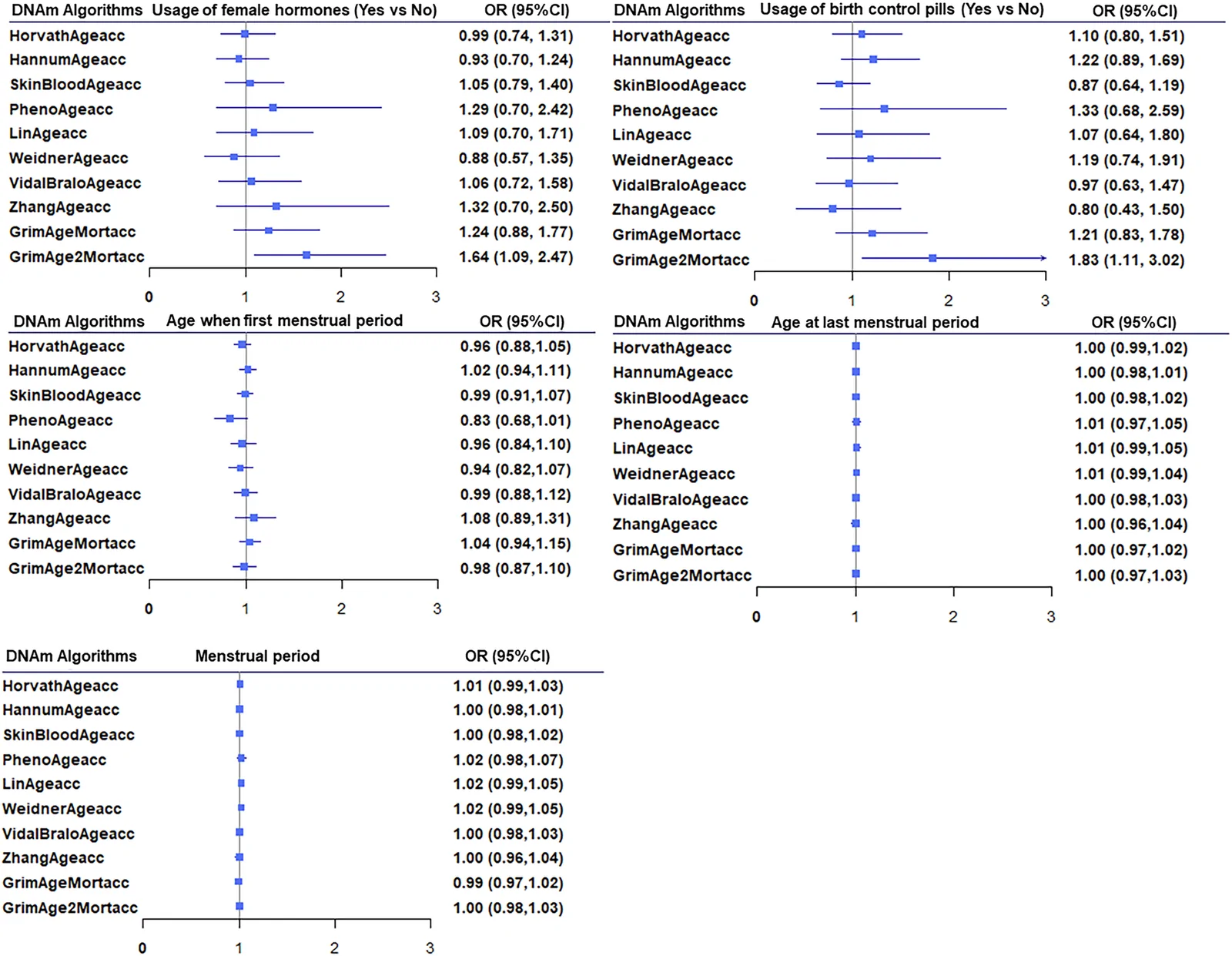
Associations of DNAm age acceleration with hormones and menstrual history. The forest plots display odds ratios (ORs) and 95% confi dence intervals (CIs) for the association of various hormonal and menstrual history factors with ten epigenetic age acceleration algorithms.

When analyzed categorically (Fig. 2), a history of female hormone use and oral contraceptive use were associated with 1.64-fold (95% CI: 1.09–2.47, *P*_FDR_ < 0.05) and 1.83-fold (95% CI: 1.11–3.02, *P*_FDR_ < 0.05) increased odds of GrimAge2Mortacc acceleration, respectively. None of the menstrual variables, including age at first menstrual period, age at last menstrual period, or menstrual years, showed statistically significant associations across the ten DNAm algorithms examined.

## Discussion

In this population-based study of 1117 women, we systematically examined the relationship between reproductive life history and epigenetic aging through a comprehensive analysis of 12 widely used DNAm aging algorithms. Our findings demonstrate that higher parity and an earlier age at first birth are associated with specific DNAm-based aging measures. Notably, the most robust links were observed with mortality-related clocks (GrimAge/GrimAge2) and phenotypic aging indices (PhenoAge), whereas several other clocks showed null or weak associations.

The observed variability across the 12 clocks suggests that biological aging is a “mosaic” process rather than a monolithic one. We speculate that these divergent associations stem from the distinct biological constructs each algorithm captures rather than mere stochastic noise. First-generation clocks (e.g., HorvathAge, HannumAge) were primarily trained to track chronological age through cellular “ticking”. In contrast, second-generation clocks (e.g., GrimAge2, PhenoAge) were developed to predict clinical biomarkers of physiological dysregulation and mortality risk^10^. Furthermore, some bidirectional patterns, where factors show protective effects for certain clocks but detrimental effects for others, highlight the complexity of reproductive hormones^11,29^.

While our findings identify clear associations, the underlying biological pathways remain speculative as they were not directly measured in this dataset (e.g., cytokines or mitochondrial markers). Drawing from external literature and animal models, we propose that these associations reflect the “physiological cost of reproduction”. Pregnancy entails significant immune reorganization and systemic inflammation (often termed “inflammaging”), which can leave stable epigenetic imprints on leukocytes^30,31^. Furthermore, the high metabolic demand of gestation and lactation induces oxidative stress and mitochondrial strain, potentially overwhelming antioxidant defenses and leading to cumulative DNA damage^32–36^. This aligns with the “disposable soma theory”, which posits that heavy resource allocation toward reproduction diverts energy from somatic maintenance and DNA repair mechanisms, a process potentially mediated by the dual role of estrogen in cell proliferation and DNA stability^37,38^. These mechanisms are supported by prior research showing accelerated telomere attrition in parous women^17,39^. Importantly, the fact that our primary associations, particularly those involving mortality-predictive algorithms, remained statistically significant after FDR correction suggests that these findings are less likely to be explained by multiple-testing alone, although they should still be interpreted cautiously.

The reproductive profile of our study population (averaging 3.0 live births with a mean age of 21 at first birth) reflects the historical reproductive norms of U.S. women born in the mid-20th century (1930s−1950s). This profile differs significantly from contemporary cohorts and may be influenced by the socioeconomic and cultural landscape of that era. To mitigate potential skewing, we adjusted for household income and education level in our multivariable models. These results suggest that the biological impact of reproductive load persists even after accounting for key socioeconomic indicators, aligning with the “weathering hypothesis” which suggests that cumulative socioeconomic and physiological stressors accelerate biological aging in specific demographic groups^28^. However, residual confounding by unmeasured economic or environmental factors remains a possibility.

To avoid oversimplification, it is important to emphasize that these relationships are not uniformly unidirectional. While the lack of association with menarche timing suggests that biological aging is not significantly altered by early-life reproductive milestones, our findings that later menopause and an extended reproductive lifespan correlate with slower aging highlight the importance of the entire reproductive window^24,37,40^. Given the relatively small effect sizes and contradictory patterns across algorithms, secondary associations should be interpreted as exploratory rather than definitive drivers of biological aging.

Several critical limitations must be acknowledged. First, the cross-sectional, observational design of the NHANES 1999 to 2002 cohort precludes causal inference, as we lack time-ordered measurements to establish whether reproductive events preceded changes in epigenetic age. Second, the reliance on retrospective, self-reported reproductive histories may introduce recall bias, particularly regarding distant life events. While likely non-differential, such measurement error could attenuate some observed associations. Third, our use of complete-case analysis resulted in the exclusion of participants with missing data, which may affect the representativeness of the final analytical sample. Fourth, while we adjusted for major sociodemographic and behavioral factors, residual confounding remains possible. Physical activity and clinical comorbidities such as cardiovascular disease and metabolic syndrome were not explicitly modeled in the primary analysis, which may influence the estimated effect sizes. Consequently, the divergent associations observed across the 12 clocks could partly result from residual confounding rather than purely distinct biological processes. While second-generation clocks such as GrimAge2 and PhenoAge are designed to capture physiological stress and mortality risk and may be more responsive to reproductive costs, unmeasured factors could affect these algorithms differently than first-generation clocks focused on chronological aging. Fifth, our mechanistic narrative remains hypothetical due to the lack of direct measurements of inflammatory biomarkers or mitochondrial function markers in this specific DNAm cohort. Sixth, the findings from this specific U.S. cohort may not be generalizable to younger generations or international populations with different reproductive norms and nutritional environments. Finally, the large number of statistical tests performed across 12 DNAm algorithms increases the risk of false positives. To mitigate this risk, we implemented FDR corrections. Although the primary associations for second-generation and mortality-related algorithms remained statistically significant after adjustment, the modest effect sizes and the exploratory nature of some secondary associations require cautious interpretation. These results should be validated in longitudinal studies that incorporate direct measures of systemic inflammation and comprehensive health records.

In summary, our study suggests that parity and reproductive timing (including age at first birth and menopausal age) are associated with biological aging in later life, though these relationships vary across different DNA methylation clocks. After adjusting for socioeconomic factors and correcting for multiple testing, the persistence of these associations indicates that reproductive history may influence epigenetic aging. However, the findings should be interpreted with caution, as no causal conclusions can be drawn. This study emphasizes the importance of using multi-algorithm approaches to better understand the complex and sex-specific aging process.

## Methods

### Study participants

Study participants were selected from the NHANES, a nationally representative, cross-sectional survey designed to assess the health and nutritional status of non-institutionalized civilians in the United States^29^. NHANES is conducted annually by the Centers for Disease Control and Prevention (CDC) through the National Center for Health Statistics (NCHS)^30^. This survey employs a complex sampling design that includes oversampling of specific subgroups to ensure a representative sample of the U.S. population^31^.

NHANES collects comprehensive demographic, health, and nutrition data from participants, who provide blood and urine samples at mobile examination centers^32^. All participants provided written informed consent prior to participation, in accordance with NCHS guidelines and the Declaration of Helsinki. This study utilized publicly available NHANES data, approved by the NCHS and CDC institutional review board (IRB). This analysis of de-identified data with no direct participant contact qualified for exemption from IRB assessment under National Institutes of Health guidelines.

**Fig. 3:**
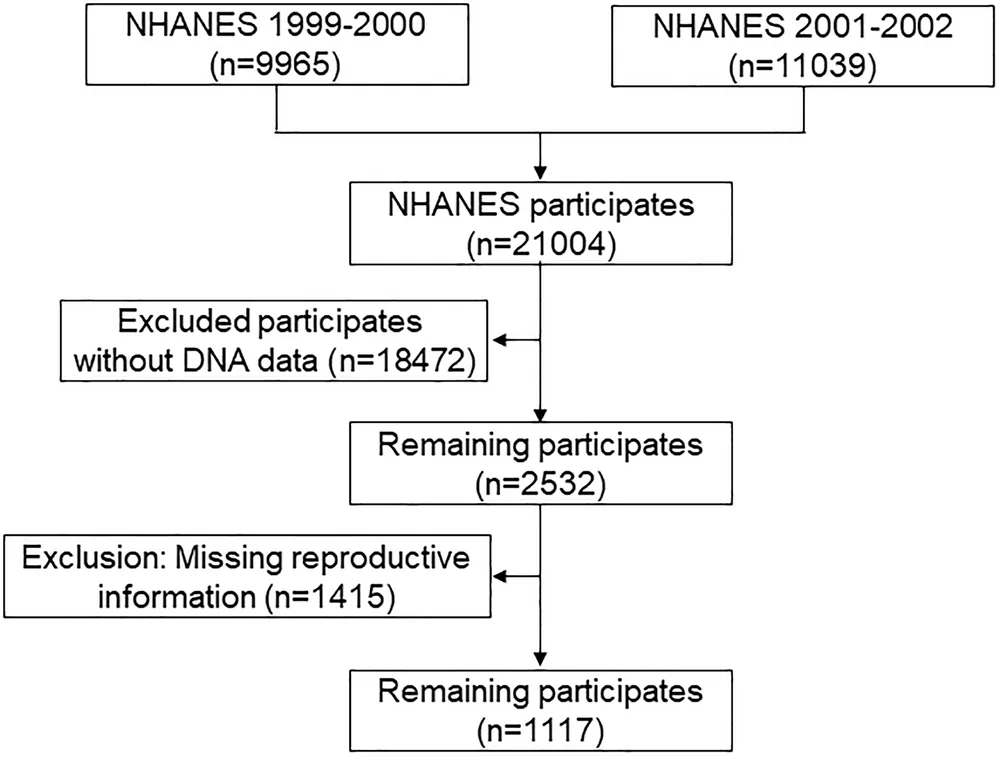
Flowchart of the participant selection process. NHANES, National Health and Nutrition Examination Survey.

For the current study, we combined data from two NHANES cycles (1999 to 2002), focusing on women aged 50 years and older. Participants were selected based on the availability of DNAm data and reproductive health information. Of the initial 21,004 participants, 18,472 were excluded because they lacked DNAm data, which was restricted to adults aged ≥ 50 years. An additional 1415 individuals were removed due to missing reproductive information, resulting in a final analytical sample of 1117 participants. The selection process is detailed in Fig. 3. Regarding the handling of missing data, a complete-case analysis was adopted for all primary multivariable models. Participants with missing values for any of the reproductive history indicators, covariates, or DNAm outcomes were excluded from the final analytical cohort. This approach was chosen to ensure the consistency of comparisons across the 12 different DNAm algorithms analyzed.

### DNAm algorithms measurements

DNAm profiling was performed by the NCHS on peripheral blood samples from adults aged ≥50 years, collected during the 1999–2002 NHANES cycles. As outlined in the NHANES DNA Methylation Array and Epigenetic Biomarkers Data Documentation^33^, DNAm was assayed using the Infinium Methylation EPIC BeadChip kit (EPIC, Illumina, San Diego, CA, USA). Pre-processing and normalization of the raw data were performed according to the manufacturer’s instructions in Dr. Yongmei Liu’s laboratory at Duke University^34^.

The NHANES epigenomics dataset includes twelve precomputed epigenetic aging biomarkers, generated using validated algorithms: (1) First-generation clocks: HorvathAge (pan-tissue)^7^, HannumAge (blood-specific)^8^, SkinBloodAge (skin-blood composite)^9^; (2) Mortality predictors: PhenoAge^10^, GrimAge (plasma protein-enhanced)^11^, GrimAge2 (updated mortality model)^35^; (3) Pace-of-aging metrics: DunedinPoAm (longitudinal decline rate)^36^, LinAge (mitotic clock)^37^; (4) Telomere length proxy: HorvathTelo (DNAm-estimated telomere length)^38^; (5) Cell-type-specific clocks: WeidnerAge (monocyte)^39^, VidalBraloAge (B-cell)^40^, ZhangAge (placental origin). These 12 epigenetic biomarkers vary in their construction processes, the number of CpG sites used, and their primary applications. A detailed comparison of their methodological and biological characteristics was described in our previous studies^41^^,42^.

To quantify epigenetic age acceleration (ΔAge), ten DNAm algorithms were computed by subtracting participants’ chronological age from their predicted biological age, except DunedinPoAm and HorvathTelo, as these directly estimate aging pace and telomere attrition without requiring residualization. The resulting ten ΔAge variables were: HorvathAgeacc, HannumAgeacc, SkinBloodAgeacc, PhenoAgeacc, LinAgeacc, WeidnerAgeacc, VidalBraloAgeacc, ZhangAgeacc, GrimAgeMortacc, and GrimAge2Mortacc.

### Reproductive health indicators ascertainment

Reproductive health indicators were selected from a self-report questionnaire based on available data for women aged 50 and older from the 1999–2000 and 2001–2002 NHANES cycles. Indicators with the potential to influence hormonal levels, reproductive health, and broader aspects of aging were included. In total, 11 reproductive health variables were included in the analysis, categorized into two groups: those related to reproductive history and those related to menstrual history:

#### Reproductive history

1. Ever been pregnant: This binary variable indicates whether a woman has ever been pregnant, providing insight into general reproductive history.
2. How many times have been pregnant: The total number of pregnancies a woman has experienced was recorded, offering a quantitative measure of reproductive events.
3. Pregnancies resulting in live births: This variable captures the total number of live births, reflecting a woman’s reproductive outcome.
4. Age at first live birth: The age at which a woman gave birth to her first child was used as an indicator of reproductive timing.
5. Age at last live birth: The age at which a woman gave birth to her most recent child was included to assess later-life reproductive activity.
6. Ever breastfed any of your children: This binary variable indicates whether a woman breastfed her children, which has been linked to various long-term health benefits.

#### Menstrual/hormonal history

1. Ever use female hormones: Data on the use of female hormones, such as estrogen or progesterone, was included to evaluate the influence of hormone therapy on health.
2. Ever taken birth control pills: Information on whether a woman ever used birth control pills was considered to assess hormonal exposure and reproductive health.
3. Age when first menstrual period occurred: The age at which a woman had her first menstrual period was included as an indicator of early reproductive development.
4. Age at last menstrual period: The age at which a woman experienced menopause was used to assess the timing of reproductive senescence.
5. Menstrual years: The number of years a woman menstruated (from menarche to menopause) was calculated, serving as an indicator of the duration of reproductive life.

### Covariates

The analytical models incorporated multiple covariates to account for potential confounding effects, guided by theoretical frameworks linking biological aging and reproductive health. These variables were selected as potential confounders associated with both reproductive history and epigenetic outcomes. To maintain theoretical consistency and avoid the limitations of automated variable selection (e.g., stepwise regression), all predetermined confounders were retained in the final models irrespective of their individual statistical significance or their effect on the primary exposure estimates. Sociodemographic characteristics including self-reported age (continuous) and highest educational attainment (categorized as <high school diploma, high school graduate, some college, or ≥bachelor’s degree) were obtained through structured interviews using validated NHANES questionnaires. To address population diversity and sampling design, race/ethnicity was classified per NHANES protocols into: non-Hispanic White, non-Hispanic Black, Mexican American, and other/multiracial groups. Economic status was represented by annual household income tiered as low (0–24,999), middle (25,000–74,999), and high (≥$75,000). Anthropometric measurements (height in cm, weight in kg) were recorded by certified examiners following CDC standardization guidelines. Body mass index (BMI) was calculated from standardized weight and height measurements.

Health behaviors, including smoking and alcohol consumption, were documented. Smoking status was categorized into three groups: Never smoker, Former smoker, and Current smoker based on the question: “Have you smoked at least 100 cigarettes in your entire life?” Alcohol consumption was categorized into three groups: Never, Ever, and Current based on the question: “In any one year, have you had at least 12 drinks of any type of alcoholic beverage?”

### Statistical analysis

Following NHANES analytic guidelines, individual data files were integrated into a unified dataset incorporating statistical weights and design elements (primary sampling units, strata) to account for complex survey methodology. Baseline population characteristics were summarized through weighted descriptive statistics, including means ± standard deviation (SD) for continuous variables and frequencies (%) for categorical measures. All descriptive and regression analyses were conducted using survey-weighted procedures that accounted for sampling weights, strata, and primary sampling units.

Multivariable linear regression models were implemented in hierarchical stages to evaluate associations between 11 reproductive health variables and 12 epigenetic aging biomarkers. Model 1 was adjusted for chronological age (continuous), leukocyte composition (estimated by the Houseman approach), and survey years. Model 2 further adjusted for BMI (continuous) and behavioral confounders, including smoking status (never/former/current) and alcohol use (never/former/current). For sensitivity analyses, epigenetic age acceleration metrics were dichotomized into accelerated (>0) versus decelerated (≤0) aging categories. Logistic regression models were then used to estimate odds ratios (ORs) with 95% confidence intervals (CIs) for reproductive health predictors across these binary outcomes, following the same hierarchical adjustment strategy.

Additional categorical analyses examined pregnancy history and live birth counts. Nulliparity (0 pregnancies) was used as the reference for gravidity comparisons, while multiparity thresholds (zero, one, two, three, four, ≥five live births) were analyzed through multinomial logistic models. To address multiple-testing concerns across 12 clocks and 11 factors, we applied the Benjamini–Hochberg FDR correction. Adjusted *P*-values (referred to as *P*_FDR_) < 0.05 were considered statistically significant.

All analyses were performed using SAS, version 9.4 (SAS Institute, Inc., Cary, NC). Statistical significance was defined by *P* < 0.05 in two-sided testing.

## Supplementary information

DNAm Women health SI R2

## Data Availability

The data used in this study are publicly available from the National Health and Nutrition Examination Survey (NHANES) program. These de-identified data can be accessed through the publicly available NHANES database.
