# Supplementary material for: Reproductive life events and biological aging in women over 50: evidence from DNA methylation clocks
Source: NPJ Aging. 2026 May 13;12(1):146. doi: 10.1038/s41514-026-00394-6 (PMC13687541; doi:10.1038/s41514-026-00394-6)
Supplement: Supplementary file 1 [file 41514_2026_394_MOESM1_ESM.docx]

**Supporting Information**

**Reproductive Life Events and Biological Aging in Women over 50: Evidence from DNA Methylation Clocks**

Ya-Qian Xu^1, 2#^, Jinming Fu^3, 4#^, Chongyu Ding^1^, Hui Zhang^1, 2^, Yulu Gong^1, 2, 5^, Darong Hao^1, 5^, Xiao Sun^6^, Ping Zeng ^3, 4*^, Xiangwei Li^1, 2*^

1. School of Global Health, Chinese Centre for Tropical Diseases Research, Shanghai Jiao Tong University School of Medicine, Shanghai 200025, China.
2. Hainan International Medical Center, Shanghai Jiao Tong University School of Medicine, Hainan 571434, China.
3. Department of Biostatistics, Jiangsu Engineering Research Center of Biological Data Mining and Healthcare Transformation, Xuzhou Medical University, Xuzhou, Jiangsu, 221004, China.
4. Jiangsu Key Laboratory of Geriatric Precision Medicine and Aging Intervention, Xuzhou Medical University, Xuzhou, Jiangsu, 221004, China.
5. School of Public Health, Shanghai Jiao Tong University School of Medicine, Shanghai 200025, China.
6. Nanyang Centre for Public Administration, Nanyang Technology University, Singapore 639798, Singapore.

**Running title**: Reproductive Health and DNAm Aging Algorithms

**^*^ Correspondence:** Xiangwei Li, e-mail: li.xiangwei@sjtu.edu.cn, Tel: +86 21-63846590-776408. Ping Zeng, email: zpstat@xzhmu.edu.cn, Tel: +86 0516-83263616

**Word count:** 340 in the abstract; 4000 in the main text.

Number of pages: 8

Number of tables: 6

**Table of Contents**

**Table Captions**

Table S1. Twelve epigenetic biomarkers of aging and their epigenetic age acceleration metrics, reproductive history, and menstrual/hormonal history.

Table S2. Odds ratio of HorvathAgeacc with number of pregnant times and number of live births.

Table S3. Odds ratio of SkinBloodAgeacc with number of pregnant times and number of live births.

Table S4. Odds ratio for PhenoAgeacc with number of pregnant times and number of live births.^a^

Table S5. Odds ratio of WeidnerAgeacc with number of pregnant times and number of live births.

Table S6. Odds ratio of ZhangAgeacc with number of pregnant times and number of live births.

**Table S1.** **Twelve epigenetic biomarkers of aging and their epigenetic age acceleration metrics, reproductive factors, and menstrual/hormonal history.**

| **Biomarkers (mean ± SD)** | **50-65 years** | **≥65 years** | **Total** |
| --- | --- | --- | --- |
| **Epigenetic biomarkers** |  |  |  |
| HorvathAge | 59.68±6.31 | 71.98±7.54 | 65.92±9.29 |
| HannumAge | 58.87±6.68 | 72.55±7.62 | 65.82±9.91 |
| SkinBloodAge | 56.59±6.38 | 70.61±7.23 | 63.71±9.78 |
| PhenoAge | 48.12±7.71 | 61.59±9.16 | 54.96±10.83 |
| LinAge | 48.47±8.07 | 64.43±10.40 | 56.57±12.28 |
| WeidnerAge | 49.09±7.85 | 59.31±10.65 | 54.27±10.68 |
| VidalBraloAge | 55.94±4.94 | 62.98±6.51 | 59.51±6.77 |
| ZhangAge | 63.68±2.41 | 69.36±2.78 | 66.56±3.86 |
| GrimAgeMort | 58.14±5.52 | 70.15±6.22 | 64.24±8.41 |
| GrimAge2Mort | 64.71±5.86 | 75.87±6.34 | 70.38±8.27 |
| DunedinPoAm | 1.09±0.09 | 1.09±0.08 | 1.10±0.09 |
| HorvathTelo | 6.80±0.25 | 6.48±0.26 | 6.64±0.30 |
| **Epigenetic age acceleration metrics** |  |  |  |
| HorvathAgeacc | 2.49±5.25 | -1.90±6.04 | 0.26±6.07 |
| HannumAgeacc | 1.68±5.62 | -1.32±5.97 | 0.16±5.99 |
| SkinBloodAgeacc | -0.60±4.79 | -3.27±5.16 | -1.95±5.16 |
| PhenoAgeacc | -9.08±6.83 | -12.28±7.20 | -10.70±7.20 |
| LinAgeacc | -8.72±6.84 | -9.45±8.28 | -9.09±7.61 |
| WeidnerAgeacc | -8.10±7.72 | -14.57±9.69 | -11.39±9.35 |
| VidalBraloAgeacc | -1.25±5.34 | -10.90±6.25 | -6.15±7.56 |
| ZhangAgeacc | 6.49±3.34 | -4.52±4.72 | 0.90±6.86 |
| GrimAgeMortacc | 0.95±4.53 | -3.73±4.05 | -1.43±4.89 |
| GrimAge2Mortacc | 7.52±5.08 | 2.00±4.60 | 4.71±5.58 |
| **Reproductive history** |  |  |  |
| Ever been pregnant (N=1,044) |  |  |  |
| Yes | 497 (95.95) | 474 (90.11) | 971 (93.01) |
| No | 21 (4.05) | 52 (9.89) | 73 (6.99) |
| Number of pregnant times (N=1,043) | 4.00 (2.00-6.00) | 4.00 (2.00-6.00) | 4.00 (2.00-6.00) |
| Number of live births (N=970) | 3.00 (2.00-5.00) | 3.00 (2.00-5.00) | 3.00 (2.00-5.00) |
| Age at first live birth (N=838) | 21.00 (18.00-24.00) | 21.00 (19.00-24.00) | 21.00 (18.00-24.00) |
| Age at last live birth (N=942) | 30.00 (25.00-34.00) | 32.00 (28.00-37.00) | 31.00 (27.00-36.00) |
| Breastfed (N=942) |  |  |  |
| Yes | 254 (51.94) | 273 (60.00) | 527 (55.83) |
| No | 235 (48.06) | 182 (40.00) | 417 (44.17) |
| **Menstrual/hormonal history** |  |  |  |
| Usage of female hormones (N=1,040) |  |  |  |
| Yes | 252 (48.93) | 202 (38.48) | 454 (43.65) |
| No | 263 (51.07) | 323 (61.52) | 586 (56.35) |
| Usage of birth control pills (N=1,042) |  |  |  |
| Yes | 347 (66.99) | 120 (22.90) | 467 (44.82) |
| No | 171 (33.01) | 404 (77.10) | 575 (55.18) |
| Age when first menstrual period | 12.86±1.76 | 13.02±1.66 | 12.92±1.71 |
| Age at last menstrual period | 44.24±8.37 | 46.24±7.90 | 45.30±8.18 |
| Menstrual years | 31.41±8.43 | 33.22±7.89 | 32.37±8.20 |

Abbreviations: SD, standard deviation.

**Table S2.** **Odds ratio of HorvathAgeacc with number of pregnant times and number of live births.**

| **HorvathAgeacc** |  | **Model 1** | **Model 2** |
| --- | --- | --- | --- |
|  | **Cases (n/N)** | **OR (95% CI)** | **OR (95% CI)** |
| **Number of pregnant times** | 1043 |  |  |
| Zero | 73 | Ref | Ref |
| One | 79 | 0.89 (0.43, 1.88) | 0.85 (0.40, 1.79) |
| Two | 142 | 1.02 (0.53, 1.96) | 0.98 (0.50, 1.91) |
| Three | 190 | 0.83 (0.45, 1.55) | 0.82 (0.44, 1.54) |
| Four | 176 | 0.85 (0.46, 1.60) | 0.83 (0.44, 1.55) |
| ≥Five | 383 | 0.84 (0.48, 1.50) | 0.80 (0.45, 1.44) |
| *P*-trend |  | 0.512 | 0.429 |
| **Number of live births** | 970 |  |  |
| Zero | 26 | Ref | Ref |
| One | 106 | 1.39 (0.51, 3.75) | 1.35 (0.50, 3.75) |
| Two | 210 | 1.62 (0.63, 4.14) | 1.63 (0.64, 4.28) |
| Three | 198 | 1.52 (0.59, 3.89) | 1.53 (0.60, 4.04) |
| Four | 140 | 1.36 (0.52, 3.55) | 1.36 (0.52, 3.65) |
| ≥Five | 290 | 1.38 (0.55, 3.48) | 1.36 (0.55, 3.54) |
| *P*-trend |  | 0.809 | 0.781 |

Abbreviations: OR, odds ratio; CI, confidence interval.

Model 1, adjusted for age, leukocyte composition (CD8TPP、CD4TPP、Nkcell、Bcell、MonoPP、NeuPP).

Model 2, similar as model 1, additionally adjusted for BMI, smoking status (never smoker, former smoker, current smoker), and drinking status (never, ever, current).

**Table S3. Odds ratio of SkinBloodAgeacc with number of pregnant times and number of live births.**

| **SkinBloodAgeacc** |  | **Model 1** | **Model 2** |
| --- | --- | --- | --- |
|  | **Cases (n/N)** | **OR (95% CI)** | **OR (95% CI)** |
| **Number of pregnant times** | 1043 |  |  |
| Zero | 73 | Ref | Ref |
| One | 79 | 0.67 (0.31, 1.44) | 0.65 (0.30, 1.40) |
| Two | 142 | 0.76 (0.39, 1.48) | 0.75 (0.38, 1.47) |
| Three | 190 | 0.65 (0.35, 1.24) | 0.65 (0.34, 1.25) |
| Four | 176 | 1.17 (0.63, 2.18) | 1.15 (0.62, 2.17) |
| ≥Five | 383 | 1.00 (0.56, 1.78) | 0.96 (0.54, 1.75) |
| *P*-trend |  | 0.142 | 0.187 |
| **Number of live births** | 970 |  |  |
| Zero | 26 | Ref | Ref |
| One | 106 | 1.72 (0.52, 5.70) | 1.63 (0.53, 6.18) |
| Two | 210 | 1.95 (0.62, 6.16) | 1.89 (0.66, 6.90) |
| Three | 198 | 2.31 (0.73, 7.33) | 2.24 (0.77, 8.17) |
| Four | 140 | 2.51 (0.78, 8.08) | 2.41 (0.82, 8.90) |
| ≥Five | 290 | 2.76 (0.88, 8.64) | 2.62 (0.92, 9.47) |
| *P*-trend |  | 0.014 | 0.021 |

Abbreviations: OR, odds ratio; CI, confidence interval.

Model 1, adjusted for age, leukocyte composition (CD8TPP、CD4TPP、Nkcell、Bcell、MonoPP、NeuPP).

Model 2, similar as model 1, additionally adjusted for BMI, smoking status (never smoker, former smoker, current smoker), and drinking status (never, ever, current).

**Table S4. Odds ratio for PhenoAgeacc with number of pregnant times and number of live births.^a^**

| **PhenoAgeacc** |  | **Model 1** | **Model 2** |
| --- | --- | --- | --- |
|  | **Cases (n/N)** | **OR (95% CI)** | **OR (95% CI)** |
| **Number of pregnant times** | 1043 |  |  |
| Zero | 73 | Ref | Ref |
| One | 79 | 0.42 (0.07, 2.06) | 0.44 (0.09, 2.27) |
| Two | 142 | 0.51 (0.13, 2.03) | 0.48 (0.13, 1.77) |
| Three | 190 | 0.39 (0.10, 1.56) | 0.36 (0.10, 1.35) |
| Four | 176 | 0.60 (0.17, 2.24) | 0.57 (0.17, 1.96) |
| ≥Five | 383 | 0.80 (0.28, 2.74) | 0.80, 0.27, 2.40) |
| *P*-trend |  | 0.329 | 0.338 |

a: The odds ratio for PhenoAgeacc with the number of live births was not available due to the small sample size in each categorical variable where PhenoAgeacc > 0 (n=49).

Abbreviations: OR, odds ratio; CI, confidence interval.

Model 1, adjusted for age, leukocyte composition (CD8TPP、CD4TPP、Nkcell、Bcell、MonoPP、NeuPP).

Model 2, similar as model 1, additionally adjusted for BMI, smoking status (never smoker, former smoker, current smoker), and drinking status (never, ever, current).

**Table S5. Odds ratio of WeidnerAgeacc with number of pregnant times and number of live births.**

| **WeidnerAgeacc** |  | **Model 1** | **Model 2** |
| --- | --- | --- | --- |
|  | **Cases (n/N)** | **OR (95% CI)** | **OR (95% CI)** |
| **Number of pregnant times** | 1043 |  |  |
| Zero | 73 | Ref | Ref |
| One | 79 | 0.31 (0.09, 1.02) | 0.30 (0.08, 0.97) |
| Two | 142 | 0.46 (0.18, 1.16) | 0.45 (0.18, 1.17) |
| Three | 190 | 0.60 (0.26, 1.39) | 0.58 (0.25, 1.40) |
| Four | 176 | 0.82 (0.36, 1.87) | 0.81 (0.36, 1.90) |
| ≥Five | 383 | 0.47 (0.22, 1.04) | 0.48 (0.22, 1.10) |
| *P*-trend |  | 0.387 | 0.453 |
| **Number of live births** | 970 |  |  |
| Zero | 26 | Ref | Ref |
| One | 106 | 1.21 (0.14, 10.90) | 1.30 (0.19, 25.87) |
| Two | 210 | 2.63 (0.33, 21.19) | 2.76 (0.50, 51.84) |
| Three | 198 | 2.82 (0.35, 22.88) | 2.96 (0.53, 55.93) |
| Four | 140 | 2.85 (0.35, 23.45) | 2.95 (0.51, 56.26) |
| ≥Five | 290 | 2.18 (0.27, 17.60) | 2.32 (0.41, 43.93) |
| *P*-trend |  | 0.604 | 0.586 |

Abbreviations: OR, odds ratio; CI, confidence interval.

Model 1, adjusted for age, leukocyte composition (CD8TPP、CD4TPP、Nkcell、Bcell、MonoPP、NeuPP).

Model 2, similar as model 1, additionally adjusted for BMI, smoking status (never smoker, former smoker, current smoker), and drinking status (never, ever, current).

**Table S6. Odds ratio of ZhangAgeacc with number of pregnant times and number of live births.**

| **ZhangAgeacc** |  | **Model 1** | **Model 2** |
| --- | --- | --- | --- |
|  | **Cases (n/N)** | **OR (95% CI)** | **OR (95% CI)** |
| **Number of pregnant times** | 1043 |  |  |
| Zero | 73 | Ref | Ref |
| One | 79 | 1.17 (0.21, 6.51) | 1.32 (0.23, 7.48) |
| Two | 142 | 0.58 (0.11, 3.25) | 0.61 (0.10, 3.48) |
| Three | 190 | 0.90 (0.23, 3.49) | 0.96 (0.24, 3.79) |
| Four | 176 | 1.79 (0.49, 6.57) | 1.84 (0.48, 6.89) |
| ≥Five | 383 | 1.95 (0.57, 6.63) | 2.07 (0.59, 7.24) |
| *P*-trend |  | 0.063 | 0.068 |
| **Number of live births** | 970 |  |  |
| Zero | 26 | Ref | Ref |
| One | 106 | 2.22 (0.22, 22.08) | 2.14 (0.21, 24.50) |
| Two | 210 | 2.66 (0.35, 20.34) | 2.49 (0.31, 23.28) |
| Three | 198 | 3.81 (0.53, 27.58) | 3.76 (0.50, 33.65) |
| Four | 140 | 4.70 (0.64, 34.65) | 4.44 (0.59, 40.08) |
| ≥Five | 290 | 5.15 (0.74, 35.65) | 4.96 (0.70, 42.89) |
| *P*-trend |  | 0.055 | 0.065 |

Abbreviations: OR, odds ratio; CI, confidence interval.

Model 1, adjusted for age, leukocyte composition (CD8TPP、CD4TPP、Nkcell、Bcell、MonoPP、NeuPP).

Model 2, similar as model 1, additionally adjusted for BMI, smoking status (never smoker, former smoker, current smoker), and drinking status (never, ever, current).
